# Understanding the multifaceted incorporation of a Zn-maize cob nanoparticle composite coating of mild steel: anti-wear, anti-corrosion, and oxidation resistance

**DOI:** 10.1039/d3ra06067e

**Published:** 2023-12-12

**Authors:** A. O. Ezzat, I. E. Ohiemi, V. S. Aigbodion

**Affiliations:** a Department of Chemistry, College of Sciences, King Saud University Riyadh 11451 Saudi Arabia; b National Research Centre of Pumps, Jiangsu University Zhenjiang 212013 China; c Africa Centre of Excellence, ACE-SPED University of Nigeria Nsukka Nigeria; d Department of Metallurgical and Materials Engineering, University of Nigeria Nsukka Nigeria; e Faculty of Engineering and Built Environment, University of Johannesburg P. O. Box 534 Auckland Park South Africa victor.aigbodion@unn.edu.ng

## Abstract

Zinc coating is one of the oldest types of coating for mild steel. The main drawback of zinc coating is that the steel loses its zinc ion after being exposed to environmental factors, leaving mild steel vulnerable to corrosion. Researchers have been working hard to create a zinc-based coating using co-deposition with agricultural waste. The outcome of this co-deposition is increased wear resistance, increased hardness, improved oxidation stability, and improved corrosion resistance. This work aims to enhance the oxidation, wear, and corrosion resistance of mild steel by co-deposition of zinc and maize cob ash nanoparticles. 0, 5, 10, and 15 wt% maize cob ash nanoparticles were used in the production of the coating. Scanning electron microscopy was used to characterize the materials' microstructure. The resulting coated samples' hardness, wear, oxidation, and corrosion properties were examined. The hardness parameters increased by 74.89% and the protection against corrosion by 76.6%. It has been shown that mild steel may have its corrosion, wear, and oxidation resistance increased by using 15 wt% maize cob ash particles.

## Abstract 2

Zinc coating is one of the oldest types of coating for mild steel.

## Graphical abstract

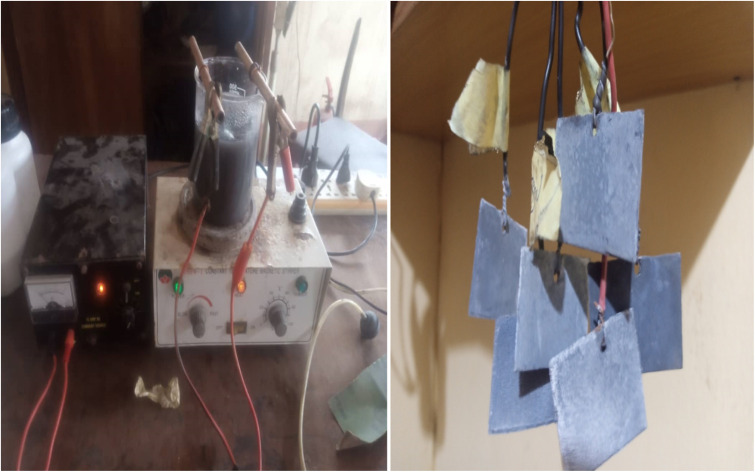

## 1 Introduction

The inadequacies of the surface features of mild steel have been addressed by protective coatings consisting of hard chromium and cadmium.^1^ Despite the fact that hard chromium and cadmium coatings have exceptional qualities like low cost and strong corrosion resistance, the electrodeposition process for these coatings is strictly regulated due to the presence of significant risks to both human health and the environment.^3,4^ According to Popoola *et al.*,^5^ cyanide plating solutions, which are very toxic to both human and animal life, are often used in cadmium plating. Chromic acid (H_2_CrO_4_), a chemical used in hard chromium electrodeposition, is also very toxic and carcinogenic.^6^

Numerous studies have been conducted in an effort to find new designs, substitute materials, and alternative procedures that do not use these hazardous substances. According to technical reports developed by the National Defense Center for Environmental Excellence, Pennsylvania electrodeposition of zinc and zinc alloys may be used to replace cadmium coating in automotive and aerospace applications.^7^ According to Shourgeshty *et al.*,^7^ the zinc-nickel alloy has better wear resistance than cadmium, and the zinc-cobalt alloy works well as a surface protector when used in SO_2_ environments.^8^ The conventional hard chromium deposit is being replaced by pure nickel, nickel–cobalt (Ni–Co), nickel–tungsten (Ni–W), cobalt–tungsten (Co–W), and ternary or quaternary alloys.^9,10^ The main drawback of zinc coating is that it loses its zinc ion after being exposed to environmental factors, leaving mild steel vulnerable to corrosion.^11,12^ Researchers have been working hard to develop a zinc-based coating using co-deposition with agricultural waste, Al, Ni, Co, and Mg to improve mild steel's corrosion resistance. The outcome of this co-deposition is likely to be higher wear resistance, higher hardness, improved corrosion resistance, and thermal stability.^13^

Fayomi *et al.*^14^ used SnO_2_ to increase the hardness, wear, and corrosion resistance of mild steel. Praveen and Venkatesha^15^ reported on the use of Zn–TiO_2_ to enhance the wear and corrosion of mild steel. Zn–ZrO_2_ coating on mild steel has excellent corrosion and wear resistance, according to Vathsala and Venkatesha's^16^ research. Loto *et al.*^17^ used extracts of ginger, celery, and pomegranate to study the electro-deposition of zinc on mild steel.

In recent time the development of sustainable coating materials has been a study in the research community worldwide, many waste materials has been successful used as a novel coating materials for mild steel, among that researched are, Aigbodion *et al.*^1^ used ant hill to developed a protective coating for mild steel, Aigbodion^2^ developed a protective coating using rice husk ash nanoparticle, Aigbodion and Akinlabi^4^ studied zinc-eggshell particles as anti-corrosion coating of mild steel, Adams *et al.*^18^ studied the wear and hardness values of CaCO_3_ obtained from eggshell for coating of mild steel, Adams *et al.*^19^ used oyster shell to developed a protective coating of mild steel. It has also been shown that silica and silicate materials offer potentially intriguing anti-corrosive qualities to mild steel by the formation of strong SiO_2_ passive thin film at the metal interfaces.^7^ According to the literature mentioned above, by using environmentally friendly materials to coat mild steel, they observed a reduction in corrosion and wear. In order to further research in this novel area of using sustainable materials that motivate interest in the development of composite coatings of mild steel using maize cob (SiO_2_) nanoparticles, because it has been reported that maize cobs contain approximately 18 kg of maize cobs for every 100 kg of corn grain and are produced in large quantities annually throughout the world, the waste causes environmental problems as a result of the open burning of the waste. The work finds another potential use of maize cob waste in the surface coating of mild steel that will be less costly, non-toxic, sustainable, environmentally friendly, and renewable.

## 2 Materials and method

### 2.1 Materials

The maize cob was obtained in the southeast region of Nigeria. Table 1 displays the composition of the mild steel that was obtained from the Ajaokuta steel plant in Nigeria.

**Table 1:** Chemical composition of mild steel (wt%)

Elements	C	Mn	Si	P	S	Zn	Al	Ni	Fe
%	0.16	0.35	0.15	0.0031	0.021	0.034	0.005	0.075	Balance

### 2.2 Method

#### 2.2.1 Production of the maize cob ash nanoparticles (MCAp)

The MCAp was produced using the sol–gel method; the details of the method are described below. The maize cob was washed and dried, the ashing was done using a muffle furnace at an operating temperature of 750 °C for 2 hours to obtain silica. A hexadecyl trimethyl ammonium bromide (CTAB) solution was added to the produced silica and agitated for 30 min in order to control the stability and morphology of the nanoparticles. NH_4_OH was then added to the solution, stirred for 20 minutes, and aged at room temperature for 10 hours. The MCAp obtained was dried for 24 hours at 105 degrees Celsius and finally calcined in a muffle furnace for four hours at 450 degrees Celsius.

#### 2.2.2 Production of the composite coating

**Fig. 1:**
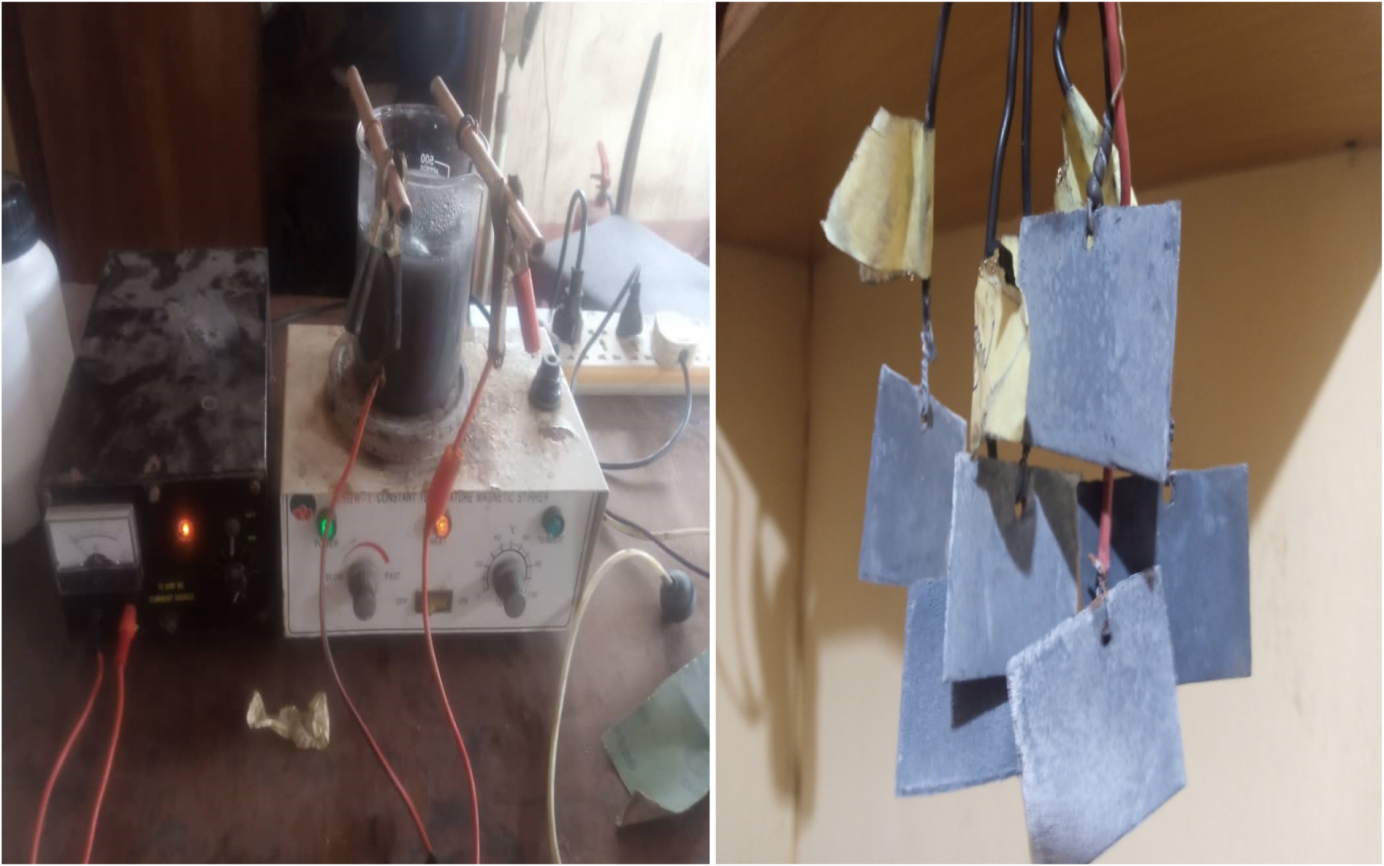
Photograph of the electrodeposition and produced sample.

Metallographic grit paper was used to ground the mild steel to remove any corroded surface, and then it was polished with alumina paste to obtain a smooth and mirror-like surface. The mild steel was cut into coupons of 4 cm by 5 cm and soaked in a 10% HCl solution for 20 minutes. The coating was produced using the co-deposition method. The deposition bath was prepared using thiourea (5 g l^−1^), ZnCl_2_ (100 g l^−1^), boric acid (15 g l^−1^), and KCl (100 g l^−1^), and 0, 5, 10, and 15 wt% MCAp were added to the bath. Preliminary experiments were conducted before choosing the composition used in the research. The solution of the bath was connected to a D.C. rectifier operating at a current density of 2.5 A cm^−2^ with the aid of an anode (zinc metal) and cathode (substrate) at a distance of 12 mm. During the electrodeposition process, the bath was stirred using a magnetic stirrer. The coating was done for 20 minutes, then removed from the bath, washed, and dried. Fig. 1 shows a photograph of the electro-deposition process.

#### 2.2.3 Characterization of the composite coated samples

The X-ray Fluorescence Analyzer Model QX 1279 was used to determine the composition of the produced MCAp. The coating thickness gauge machine type BC-770 was used to measure the thickness of the developed samples. The hardness test was carried out using a Vickers hardness tester (Matsizuwa Seiki Vickers hardness tester) at a constant load of 100 g and a dwell time of 15 seconds. An electrochemical analyzer of type CHI660D was used to determine the corrosion resistance of the samples in simulated seawater. Ag/AgCl_2_ (the reference electrode), a graphite rod (the counter electrode), and the coated sample (the working electrode) were the three electrodes used for the corrosion test. The working electrode (1.0 cm^2^) was submerged in simulated seawater (3.5% NaCl). The corrosion test was done with the ISO 9227:2017 standard, using −0.5 V to 0.5 V as the potential at a 30 °C temperature and a scanning rate of 0.5 mV s^−1^. Eqn (1) was used to compute the polarization resistance (*R*_P_).1
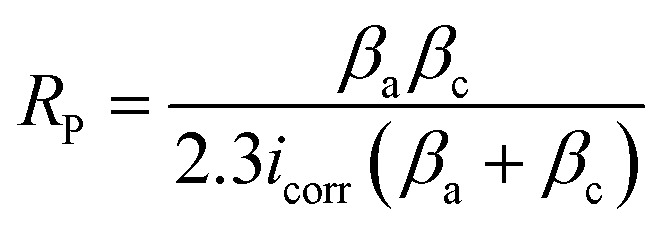
where: *β*_a_ = anodic constant, *β*_c_ = cathodic constant *i*_corr_ = current density.

The microstructure and the phases distribution of the samples were determined by scanning electron microscope model: VEGA 3 TESCAN and X-ray diffractometer model: X'Pert Pro. The wear test of the produced samples was determined using a tribometer model, RTEC 2441-USA. A wear test was done using an applied load of 100 N, a speed of 2.5 m s^−1^, and a sliding distance of 1000 N. Eqn (2) was used to compute the volumetric wear loss.2
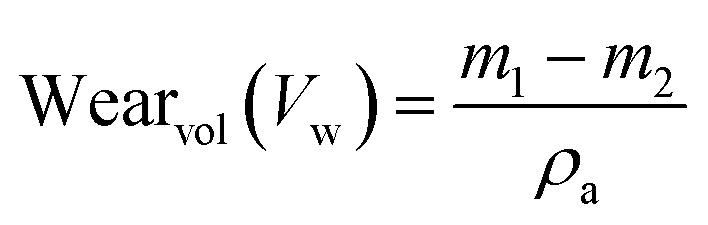
where: *m*_1_ = initial mass (g), *m*_2_ = final mass (g), *ρ*_a_ = density of samples (g cm^−3^).

## 3 Results and discussion

### 3.1 TEM image of MCAp

**Fig. 2:**
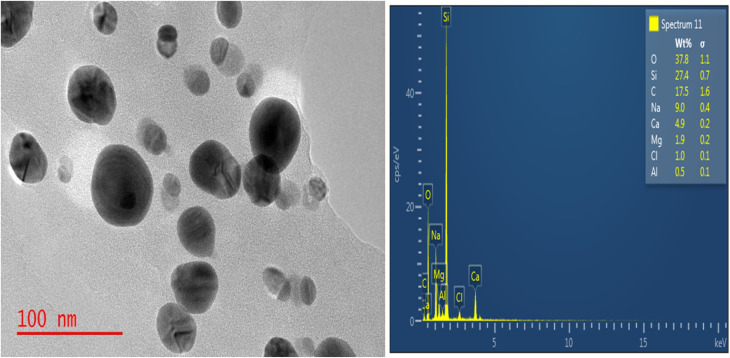
TEM image of BSAnp.

Fig. 2 displays the TEM picture of the MCAp. The dominant phases in the TEM picture were the spherical and circular features, which were easily discernible. There was no indication of particle grouping or segregation. Average particle sizes ranged from 97 to 69 nm. According to the silicon TEM image, there is a noticeable peak in the EDS. The resulting MCAp may be useful for coating applications because of the high peak of Si. This is consistent with the work of Choi *et al.*^20^

### 3.2 Chemical composition of the MCAp

Table 2 displays the results of the MCAp XRF analysis. The MCAp was found to have a 37.00% SiO_2_ content percentage as its major component. The composition of the MCAp as obtained is consistent with Ji Yong *et al.*'s^20^ analyses.

**Table 2:** Chemical composition of MCAp

CaO	SiO_2_	MgO	K_2_O	Al_2_O_3_	Na_2_O	Fe_2_O_3_	P_2_O_5_	SO_3_
10.28%	67.33%	1.82%	4.2%	7.34%	0.39	3.74%	1.04%	1.11%

### 3.3 Microstructure

**Fig. 3:**
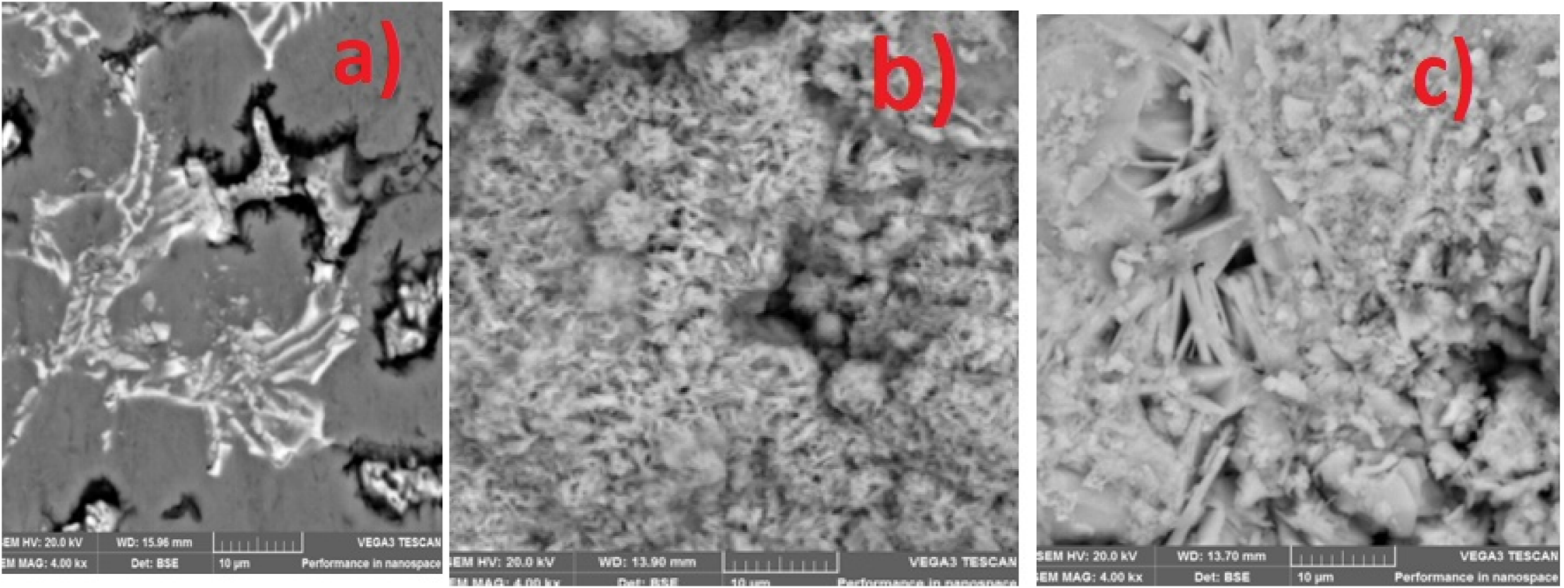
SEM of the (a) substrate (b) 5 wt% MCAp coated sample (c) 15 wt% MCAp coated sample.

**Fig. 4:**
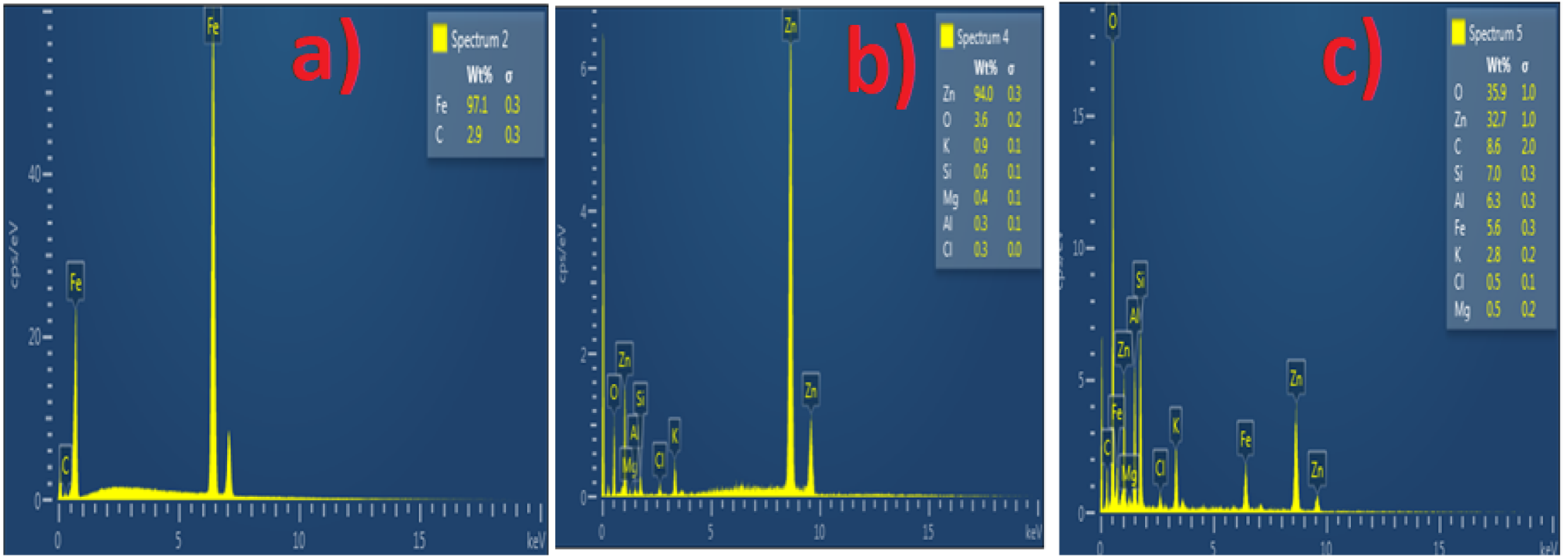
EDS of the (a) substrate (b) 5 wt% MCAp coated sample (c) 15 wt% MCAp coated sample.

The SEM and EDS of the samples are shown in Fig. 3 and 4. Fig. 3b and c demonstrated that MCAp could be seen on the samples' surfaces. Fig. 3b and c show how the developed samples microstructure changes as compared with that of the mild steel (Fig. 3a). The uncoated sample's surface appears transparent and devoid of material deposition; however, Fig. 3a makes it abundantly evident that pearlite and ferrite with high grain sizes are present. However, the coated sample's surface exhibits a thin, uniform layer of MCAp nanocrystalline nodules covering the whole surface. The higher concentration of MCAp observed in Fig. 3c was attributed to the fact that increases in dissolution of MCAp lead to globules of Zn-MCAp. The SEM displayed the strength of the second-phase bonding of MCAp as a result of the increased dissolution of MCAp. The effective coating is shown in the SEM image, which does not show any holes, fractures, or other faults. The uniform coating was caused by grain refining and a reduction in the nucleation density. The observation is in line with the work of Adams *et al.*^21^ which reported using oyster shell as a coating material results in few surface defects, smaller crystallites, and a compact and dense deposit. Utilizing an energy dispersive spectrometer (EDS), the SEM pictures were analyzed (Fig. 4). The increase in the peaks of C and Fe observed in the EDS of the substrate and the absence of Si and Zn show that the steel used is carbon steel (Fig. 4a). Fig. 4b and c revealed a robust Zn and Si signal, this shows that the coated samples that are shown in Fig. 3b and c were made from the MCAp-coated materials.

### 3.4 XRD analysis

**Fig. 5:**
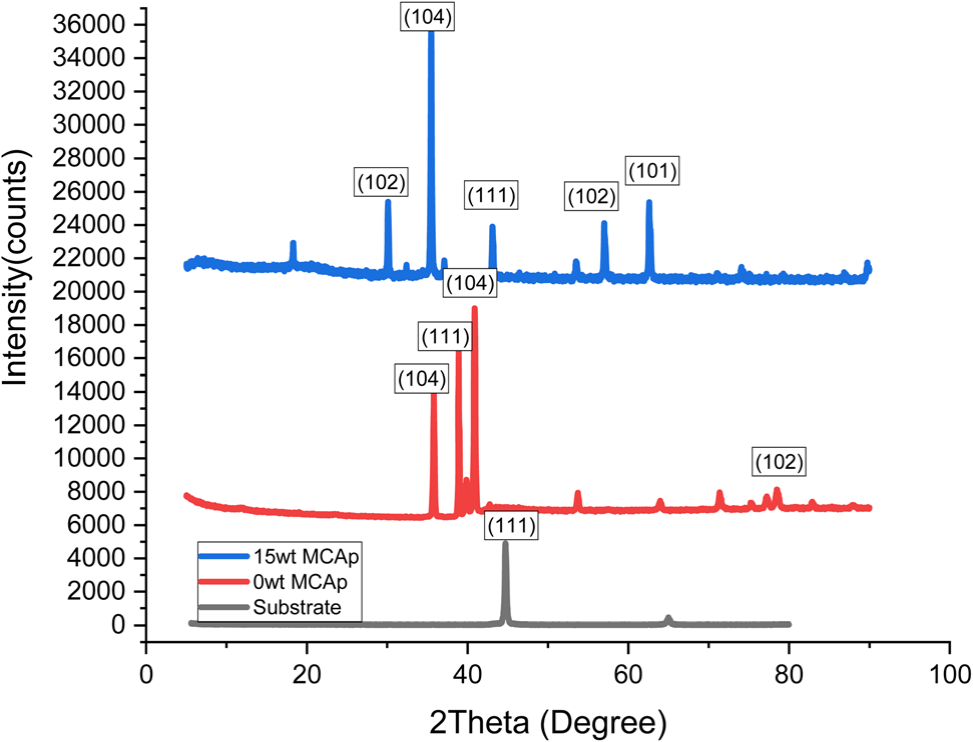
Results of XRD analysis.

The XRD spectrum of the developed composite coatings on mild steel is presented in Fig. 5. The Zn-MCAp coating exhibited a micro-crystalline structure and stable phase with distinct peaks, while the substrate only displayed two main phases of the crystallographic plane of (101) and (111) and which corresponded to C (hexagonal) and α-Fe (cubic) respectively. In addition to the C (hexagonal) and α-Fe (cubic) phases in the coated samples, there were also phases of ZnO (cubic) and SiO_2_ (Rhombohedral) phases. The 15 wt% MCAp coated sample showed evidence of more diffraction peaks sparingly observed. This was used to support the earlier conclusion that more SiO_2_ transfers to the cathode during the electrodeposition.^12^

### 3.5 Hardness values and coating thickness

**Fig. 6:**
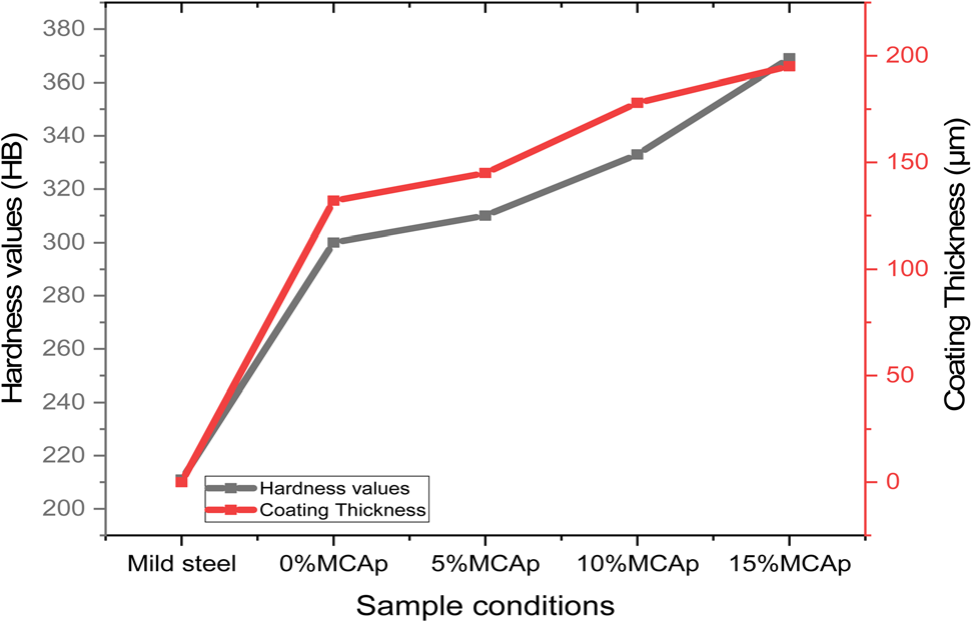
Hardness values and coating thickness with sample conditions.

Fig. 6 displays the results of the hardness values and coating thickness. It was seen that both the results of the hardness values and coating thickness values have a similar pattern, meaning that as the hardness values increase, the coating thickness also rises. This shows that there is a direct relationship between coating thickness and hardness values. This could be attributed to the fact that the MCAp covered the substrate surface, which led to rises in the high hardness values as a result of dispersive strengthening, grain refining, strain hardening, and a rise in dislocation density. A similar observation was observed in the work of Aigbodion *et al.*^1^ A 369 and 211HB were obtained as the hardness values of the coated and substrate samples respectively; this corresponded to an increase in hardness of 74.89%. A rise in hardness levels was previously observed by Zheng *et al.*^12^

### 3.6 Analyses electrochemical corrosion

**Fig. 7:**
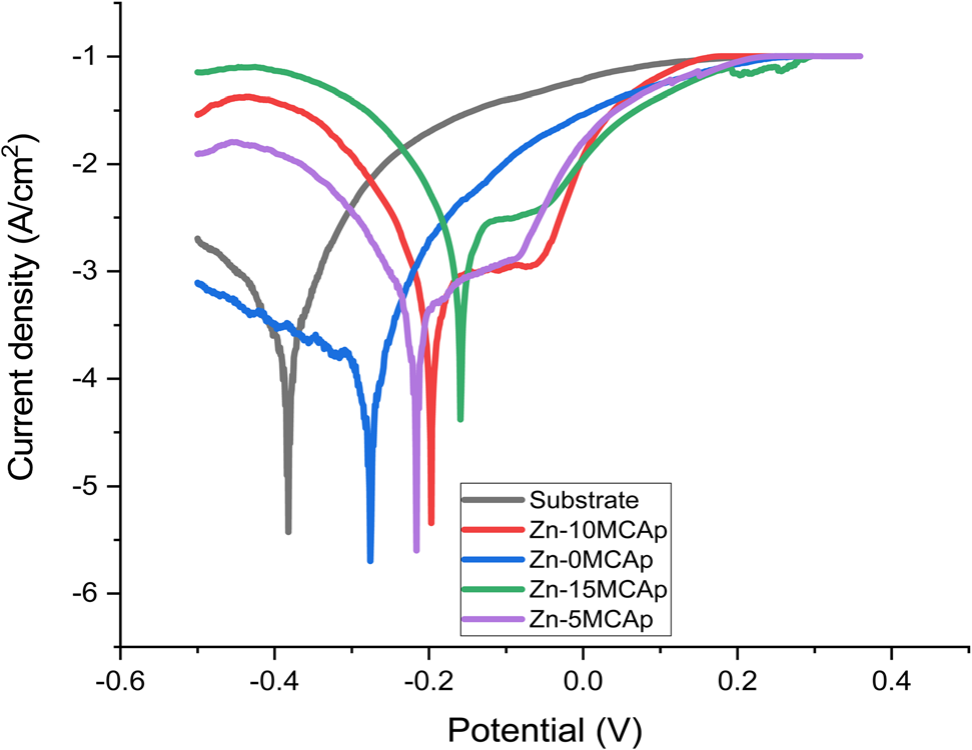
Tafel plots of the corrosion test.

**Fig. 8:**
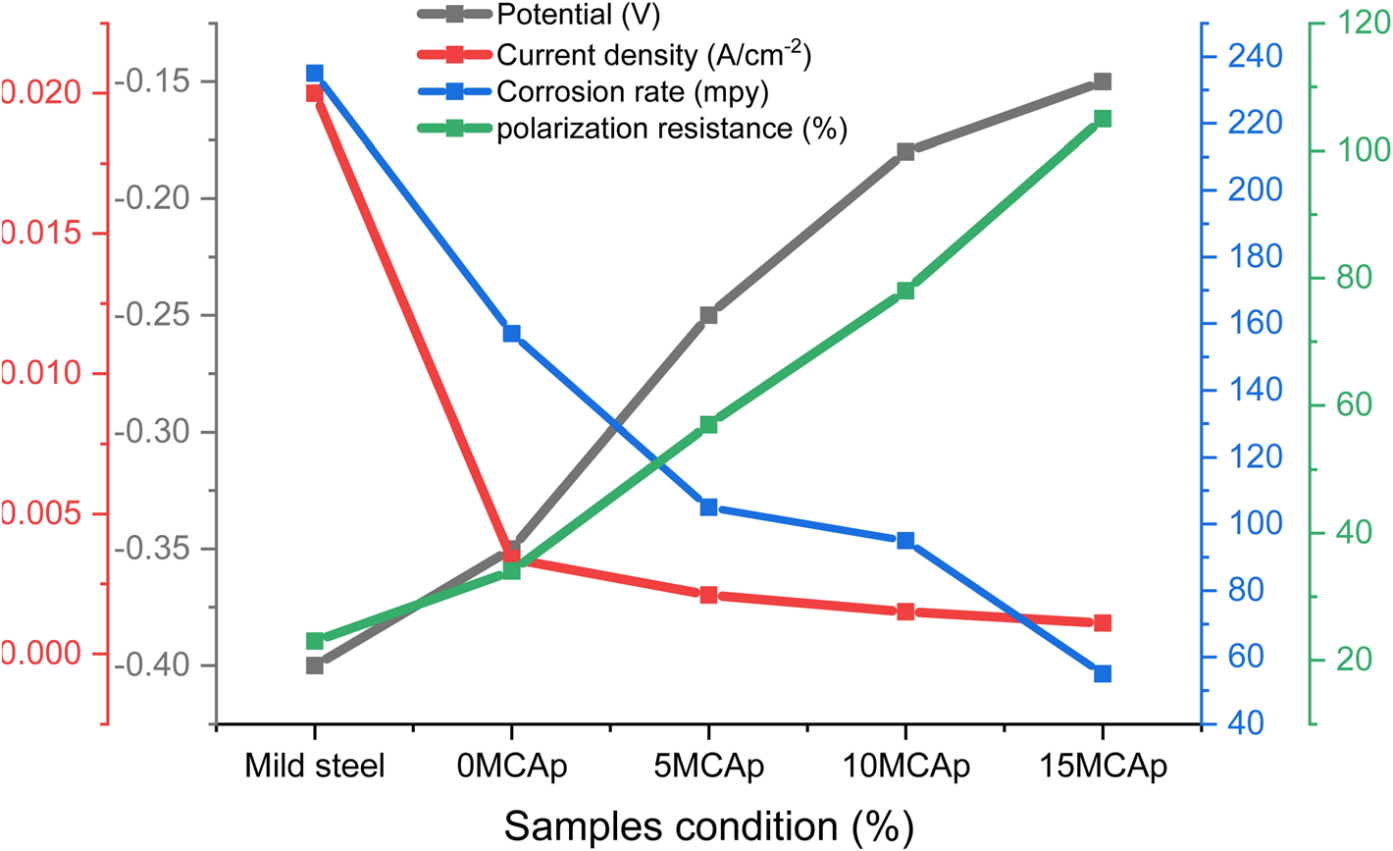
The results of current density, potential, polarization and corrosion rate with sample conditions.

Fig. 7 and 8 display the results of the Tafel graphs and corrosion properties. It was seen in Fig. 6 that the coated samples are more noble than the substrate; this shows that the MCAp helped develop a protective layer that shielded the metal from direct contact with the corrosion medium, which shifted the coated samples potential to the right, reduced current density and corrosion rate, and increased potential and polarization resistance. Mild steel dissolves more rapidly because there are more chloride ions in the mixture, as shown in eqn (3) and (4), which leverage on the unstable oxide layer as a result of the mild steel's discharge of electrons into the chloride environment. The dissolution of iron hydroxide in the medium results in a vivid, fresh solution that is produced when the iron ions mix with the hydroxide ions, as shown in eqn (5) and (6).

3 Fe → Fe^2+^ + 2e^−^

4 Fe + Cl^−^ + H_2_O → [FeCl(OH)]_ad_^−^ + H^+^ + e^−^

5 [FeCl(OH)]_ad_^−^ → FeClOH + e^−^

6 FeClOH + H^+^ = Fe^2+^ + Cl^−^ + H_2_O

There is considerable corrosion attack on the substrate as a result of the unstable oxide that peels off from the surface. The findings are consistent with the recent research of Zheng *et al.*^12^ However, in the samples that had been coated, a thick layer of permanent oxide that fully covered the mild steel's surface lowered the severity of the attack by corrosion effect, which results in lowering the level of Cl^−^ attack. Fig. 8 demonstrates that as the coated samples' polarization resistance and potential increase, the current density and rate of corrosion decrease. For instance, the corrosion rates of the samples are 235, 157, 105, 95, and 55 mpy, with a corrosion potential of −0.4, −0.35, −0.25, −0.18, and −0.15 V for the substrate, 0 wt% MCAp, 5 wt% MCAp, 10 wt% MCAp and 15 wt% MCAp respectively. This results in an increase in corrosion protection of 76.60% at 15 wt% MCAp.

**Fig. 9:**
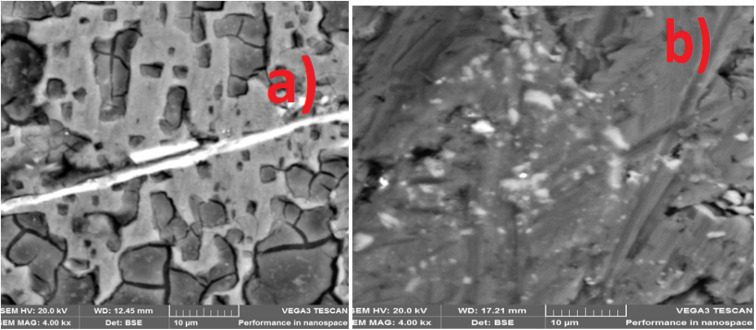
SEM image of corroded surface (a) mild steel (b) 15 wt% MCAp coated sample.

The corrosion-worn surface in Fig. 9 illustrates how the nanocrystalline nodular structure of MCAp helps in the production of a stable layer that was attributed to the high corrosion protection of the coated sample (Fig. 9b). The substrate exhibits greater corrosion damage, which is shown in Fig. 8a in the form of sizable pits and cracks. The severe corrosion damage to the substrate is to be attributed to a local corrosion attack. The local corrosion attack mechanism results from damage that preferentially occurs at specific locations on a mild steel surface is known as localized corrosion, and it may lead to the production of pits, fissures, and grooves as observed in Fig. 9a.

### 3.7 Analysis of wear rate

**Fig. 10:**
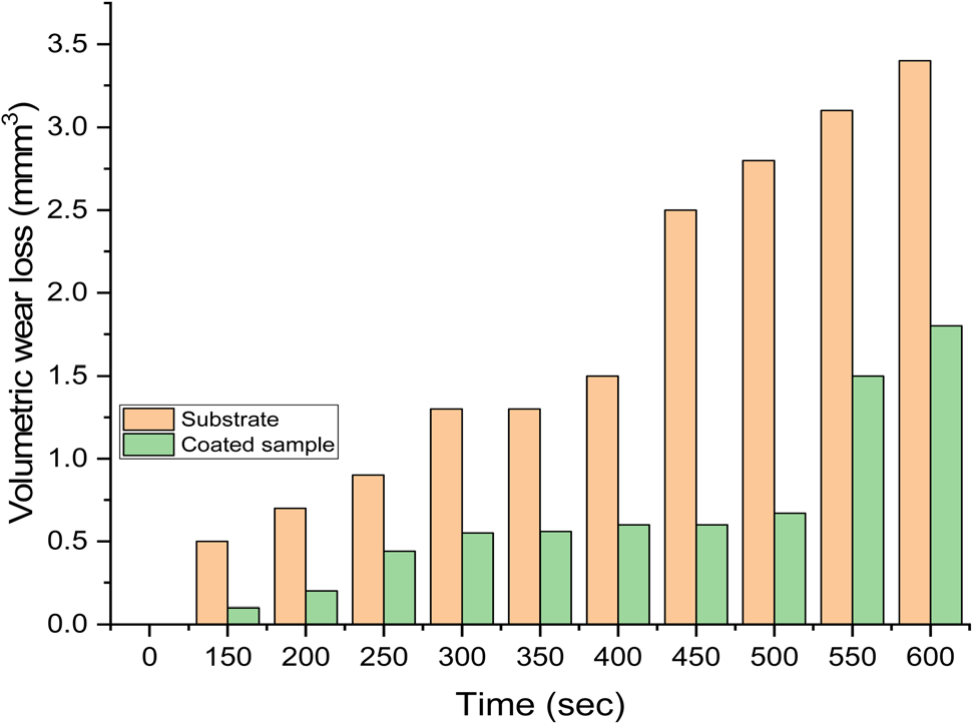
The volumetric wear rate with time.

Fig. 10 displays the results of the wear rate. It was clear that the substrate shows higher wear than the samples with Zn-15 wt% MCAp coating. The decreased wear rate was attributed to the hardness values provided by the coated sample while sliding as a result of the addition of hard MCAp in the bath formulation. A similar observation has been previously reported on the materials' ability to self-lubricate.^1^ The lubrication caused by the addition of MCAp, as demonstrated in the SEM image, helps reduce wear loss.

**Fig. 11:**
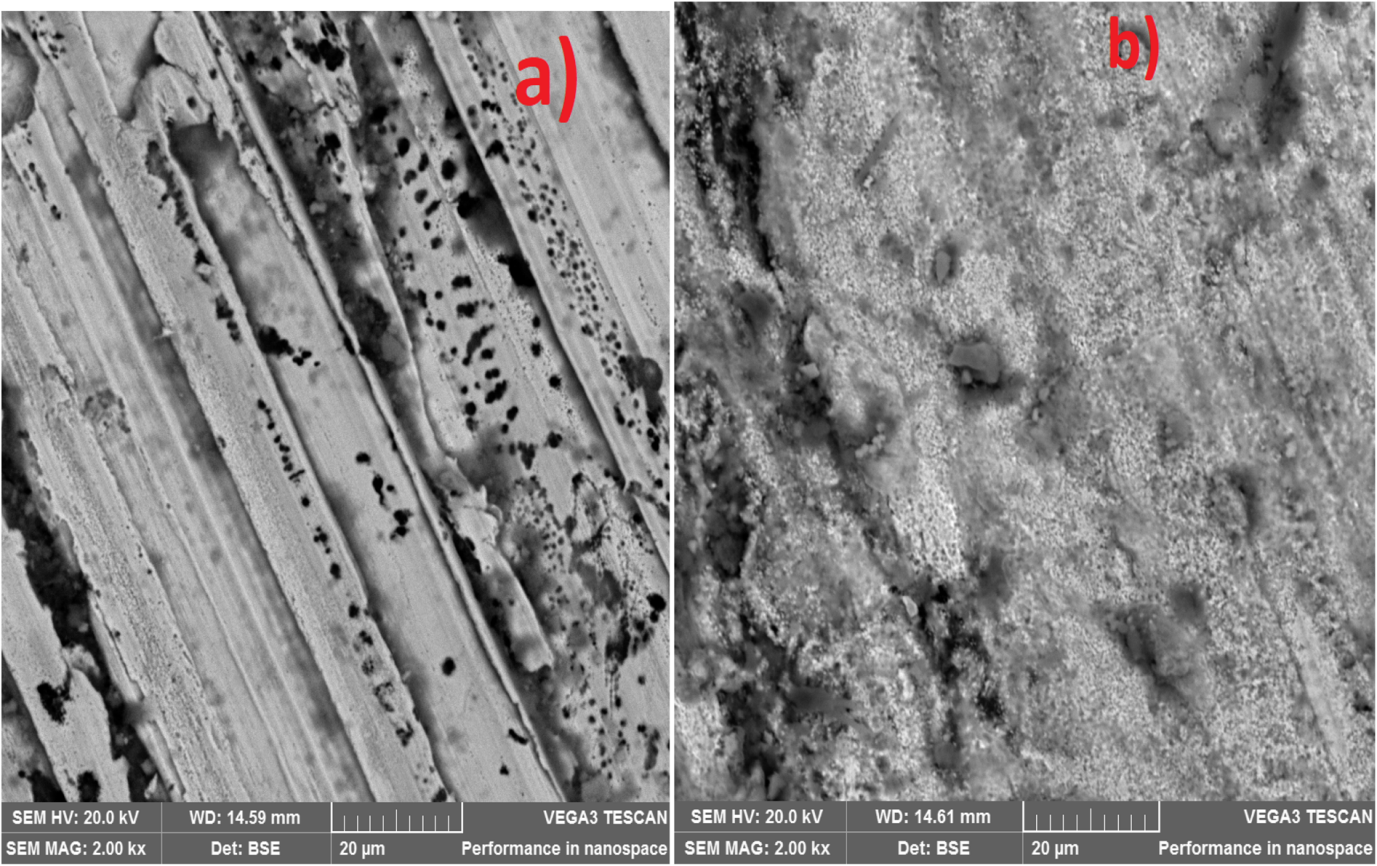
SEM image of wear worn surface (a) mild steel (b) 15 wt% MCAp coated sample.

The substrate's worn-out surface (Fig. 11a) shows high craters and grooves in comparison to the coated sample (Fig. 11b). Both adhesive and abrasive wear may be seen as results of the craters, grooves, and delaminations that formed on the substrate. The sample that has been coated showed the most reduction in wear. Adams *et al.*^21^ provided support for the lower wear rate found, stating that the low wear rate, which results in high Si atom in the coated sample help to lubricant the surface during sliding.

### 3.8 Oxidation behavior

**Fig. 12:**
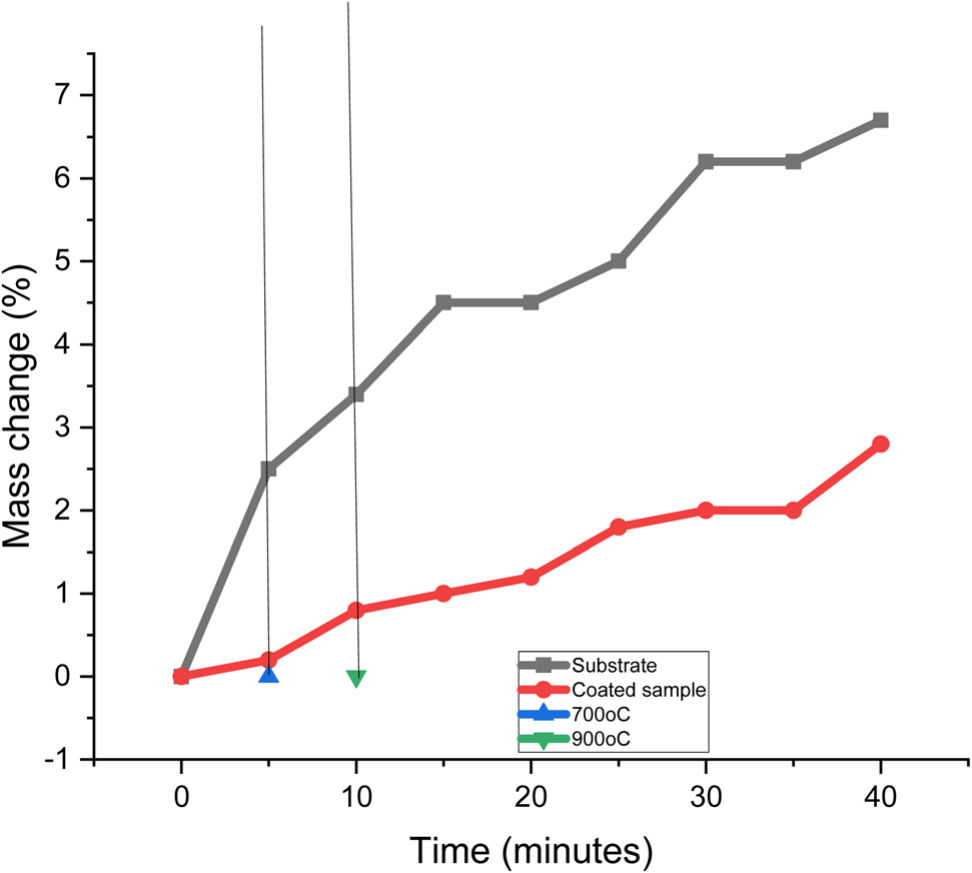
The Mass change with time.

Fig. 12 displays the oxidation properties of the samples. The two samples' oxidation patterns were quite different from one another. It took the samples ten minutes to reach their target temperature of 900 °C. The difference between the sample's original weight and the weight increased by oxidation was used to compute the mass changes. However, there was no mass change below a temperature of 700 °C. This shows that substrate undergoes decarbuzation at a higher temperature than 700 °C. During the decarburization procedure, scale was produced on the sample's surface. The coated sample is more oxidation-resistant at the higher temperature. The development of the protective layer of MCAp on the surface of the mild steel was attributed to the high oxidation resistance of the coated sample. For instance, mass fluctuations of 2.8 and 6.5% were obtained for the mass change for the coated sample with 15 wt% MCAp and substrate, which correspond to 56.92% oxidation protection of the substrate. The resistance of the coated sample to oxidation is in line with how glass and ceramic materials respond to oxidation.^11^ Furthermore, a potential oxidization prevention mechanism of the MCAp coating on mild steel has been proposed to the high temperature shielding. This mechanism takes into the formation of an interfacial phase upon heating, initial sample surface roughness and thermal expansion.^11^

## 4 Conclusions

The development of a high-performance composite coating from zinc/maize cob ash nanoparticles was investigated as a replacement for the toxic and high-cost materials used in the surface coating of mild steel. The enhancement of mild steel corrosion and microstructure was explored in the present study. The following conclusions are possible from the study:

1. Zn-maize cob ash nanoparticle coating was successfully developed on mild steel.

2. The nodule structure formed in the coating as a result of the addition of the maize cob ash nanoparticles was attributed to the rise in hardness values, corrosion resistance, and wear resistance of the substrate.

3. The hardness values, coating thickness, corrosion resistance, and wear resistance increased as the wt% MCAp increased from 0 to 15.

4. A corrosion protection of 76.6% was obtained at Zn-15 wt% maize cob ash particles.

5. The values for hardness increased by 74.889%. A rise in dislocation density is what led to this increase in hardness levels.

6. It has been shown that maize cob ash particles may be used to improve mild steel's resistance to corrosion, wear, and oxidation.

## Ethical approval

This work does not include humans and animal, hence does not require ethical approval from any committee.

## Consent to participate

This work does not include humans and animal hence does not required Consent to participate in the research.

## Consent to publish

The Authors give the publisher the consent to publish the work.

## Data availability

The authors confirm that the data supporting the findings of this study are available within the article.

## Author contributions

All authors contributed to the study conception and design. Material preparation, data collection and analysis. All authors read and approved the final manuscript.

## Conflicts of interest

There is no competing of interests to report in this work.

## Supplementary Material
